# Efficacy comparison of multi-phase CT and hepatotropic contrast-enhanced MRI in the differential diagnosis of focal nodular hyperplasia: a prospective cohort study

**DOI:** 10.1186/s12876-017-0719-1

**Published:** 2018-01-15

**Authors:** Tomasz K. Nowicki, Karolina Markiet, Ewa Izycka-Swieszewska, Katarzyna Dziadziuszko, Michal Studniarek, Edyta Szurowska

**Affiliations:** 1 2nd Departement of Radiology, Medical University of Gdansk, Smoluchowskiego 17, 80-214 Gdansk, Poland; 2 Department of Pathology and Neuropathology, Medical University of Gdansk, Debinki 1, 80-211 Gdansk, Poland; 3 Departement of Radiology, Medical University of Gdansk, Debinki 7, 80-211 Gdansk, Poland

**Keywords:** Focal nodular hyperplasia, Magnetic resonance imaging, Computed tomography, Differential diagnosis, Hepatobiliary phase, Gadobenate Dimeglumine, AFROC

## Abstract

**Background:**

Different clinical behaviour influences the importance of differentiating focal nodular hyperplasia (FNH) from other focal liver lesions (FLLs). The aim of this study was to compare the efficacy of contrast-enhanced CT and MRI in the diagnosis of FNH.

**Methods:**

157 patients with equivocal FLLs detected in ultrasonography subsequently underwent multi-phase CT and MRI with the use of hepatotropic contrast agent (Gd-BOPTA) in a 1.5 T scanner. Examinations were evaluated by three independent readers. Diagnostic efficacy of different radiological signs of FNH in both CT and MRI was compared and AFROC analysis was performed.

**Results:**

4 hepatocellular adenomas, 95 hepatocellular carcinomas, 98 hemangiomas, 138 metastases and 45 FNHs were diagnosed. In both CT and MRI the radiological sign of the highest accuracy was the presence of the central scar within FNH (0.93 and 0.96 relatively). The sum of two radiological signs in MRI: homogeneous enhancement in hepatic arterial phase (HAP) and enhancing lesion in hepatobiliary phase (HBP) was characterized with high values of sensitivity (0.89), specificity (0.97), PPV (0.82), NPV (0.98) and accuracy (0.96). After inclusion of clinical data into analysis the best discriminating feature in MRI was the presence of enhancing lesion in HBP in patients without cirrhosis. In this regard, efficacy parameters increased to 1.00, 0.99, 0.94, 1.00 and 0.99 accordingly. The area under the curve in AFROC analysis of MRI performance was significantly larger than of CT (*p* = 0.0145).

**Conclusion:**

Gd-BOPTA-enhanced MRI is a more effective method in the differential diagnosis of FNH than multi-phase CT.

**Electronic supplementary material:**

The online version of this article (10.1186/s12876-017-0719-1) contains supplementary material, which is available to authorized users.

## Background

Focal nodular hyperplasia (FNH) is a benign lesion, composed of hyperplastic hepatocytes separated by fibrous septa with common central scar (see Additional file 1). FNH is most probably a reactive proliferation of hepatocytes due to preexistent vascular malformation [1].

It is the next most frequent benign liver lesion following hemangioma and usually develops in unchanged liver parenchyma [2]. It is encountered in 0.3%–6% of the general population [3] but the incidence is increasing, partly due to progress in radiological imaging.

Different clinical behaviour and pathological features influence the importance of differentiating FNH from other hypervascular liver lesions such as hepatocellular adenoma (HCA), hepatocellular carcinoma (HCC), and hypervascular metastases as it is critical to ensure proper treatment.

Clinical symptoms and biochemical parameters in FNH are nonspecific. Diagnosis based on ultrasonography and core needle biopsy may not be conclusive. Furthermore, correct diagnosis of FNH in computed tomography (CT) and magnetic resonance imaging (MRI) may not be possible even in about 30% and 20% of cases respectively due to atypical radiological features [4, 5]. However, MRI with the use of organ-specific contrast agents optimizes diagnosis [6–9]. There are two hepatotropic contrast agents available: gadoxetic acid and gadobenate dimeglumine (Gd-BOPTA). Hepatotropic contrast agents applied in MRI have a vascular-interstitial distribution during the first few minutes and are partially excreted by kidneys to urine. 3–5% of Gd-BOPTA and 50% of the gadoxetic acid is taken up by hepatocytes with normal metabolism and secreted into bile [10]. Due to contrast agent uptake by hepatic cells, it is possible to observe the enhancement of the liver parenchyma in so-called hepatobiliary phase (HBP). Enhancement persists for 1–4 h after administration of Gd-BOPTA and 20–40 min after gadoxetic acid [11]. Lesions lacking active hepatocytes are less enhancing than surrounding parenchyma or not enhancing at all. Parenchymal cells constituting FNH accumulate hepatotropic contrast agents in opposition to hepatic hemangiomas (HH) and the majority of metastases and hepatocellular carcinomas (HCC).

To compare the efficacy of two imaging modalities (CT vs. MRI) in the assessment of multiple liver lesions in different locations a conventional receiver operating characteristic (ROC) is not sufficient. However, the alternative free-response receiver operating characteristic (AFROC) methodology incorporates lesions location and confidence level of a reader [12]. AFROC curve is a plot of the fraction of correctly diagnosed lesions in their true location (lesion location fraction, LLF) and false positive fraction (FPF) [12].

The aim of this study is to compare the efficacy of multi-phase multi-detector CT and MRI with the use of hepatotropic contrast agent Gd-BOPTA in the differentiation of FNH from other focal liver lesions (FLLs) in patients with equivocal foci detected in ultrasonography.

## Methods

**Fig. 1:**
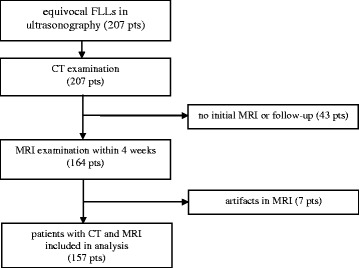
Flowchart of patients’ inclusion in the analysis. FLLs – focal liver lesions, pts – patients

In this prospective study, we included 207 patients with equivocal FLLs detected in ultrasonography and no contraindications to CT and MRI including administration of iodine contrast agent and Gd-BOPTA respectively. Multi-phase multi-detector CT and dynamic contrast-enhanced liver MRI with the use of hepatotropic contrast agent Gd-BOPTA were performed within 4 weeks. 43 patients did not show for either initial MRI or follow-up. Seven patients were excluded due to artifacts in MRI preventing further evaluation of lesions (Fig. 1).

### CT & MRI protocol

CT examination consisted of non-enhanced and contrast-enhanced phases. Iodine contrast agent was administered by power injector at the flow rate of 4 mL/s and hepatic arterial phase (HAP), portal venous phase (PVP) and equilibrium phase (EP) were acquired. In order to optimize the parameters of multiphase examination, bolus tracking technique was applied. CT section thickness was 2–2.5 mm, pitch - 1.5.

Liver MRI examination was performed with the use of a 1.5 T scanner and a phase array coil or body coil. Non-contrast examination included spin echo sequence (TR/TE – 303/12 ms, scan time – 17 s) and single shot fast spin echo sequences (18,000/80 ms, 17 s) in T1 and T2-weighted images in three perpendicular planes as well as sequences with fat saturation - fast spin echo sequences (6500/116.8 ms, 22 s) and spoiled gradient echo sequence (150/2.24 ms, 12 s, flip angle of 90°). Section thickness was 5 mm, intersection gap – 1 mm, FOV 35 × 32 cm, matrix – 256 × 256. Gd-BOPTA was administered in manual bolus through a venous catheter in a dose of 0.1 mmol/kg per body weight. HAP, PVP and EP were obtained after 25, 60 and 180 s post-contrast agent administration respectively. HBP in spoiled gradient echo and spin echo sequences was obtained 60 min after contrast agent administration. Phase-encoding direction was anterior-posterior for all sequences. All images were acquired with breath-hold technique.

### Image analysis

CT and MRI were evaluated by three independent readers with at least 5 years of experience in abdominal imaging. Readers, who were blind to patients’ clinical data and ultrasonography reports analyzed CT examination in the first session and MRI in the second session.

Number, longest diameter in axial plane and localization of lesions within liver segments, their density in CT and signal intensity on T1- and T2-weighted images were evaluated. Signal intensity was assessed in comparison to the adjacent liver parenchyma in T1- and T2-weighted images. The pattern of contrast enhancement in consecutive phases and the presence of central scar were further assessed. In HAP peripheral (ring-like or peripheral nodular enhancement) or central (homogeneous or heterogeneous) enhancement patterns were registered. In PVP enhancement pattern was divided into following subtypes: equal to the liver, ring enhancement, centripetal enhancement, homogeneous enhancement, heterogeneous enhancement or absence of enhancement (wash-out). In EP and HBP density and/or signal intensity of the lesions in comparison with the adjacent liver parenchyma was assessed.

Lastly, readers chose the most likely diagnosis and provided confidence level in both CT and MRI. The following diagnoses were proposed: HH, HCA, FNH, HCC and metastasis. The confidence level was given according to a five-point scale: 5 – very likely, 4 – likely, 3 – equivocal, 2 – unlikely, 1 – very unlikely.

### Statistical analysis

For the purpose of analysis, patients were divided into two groups according to the final diagnosis: one group consisted of patients with FNH (FNH group) and the other of patients with remaining FLLs (non-FNH group). The mean of patients age and lesion size was calculated in both groups. The χ^2^ and Mann-Whitney U tests were used for population and lesion size analysis.

To establish diagnostic criteria enabling recognition of FNH on the basis of CT and MRI, statistically more frequent radiological signs were selected in the FNH group. Additional clinical data such as lack of liver cirrhosis and neoplastic disease in anamnesis were evaluated in accordance with the definition of FNH [13]. Sensitivity, specificity, PPV, NPV and accuracy for given radiological signs and clinical features differentiating FNH from other FLLs were calculated.

Subsequently, the performance of different features was assessed by means of Cochran’s Q test and *post hoc* analysis with McNemar’s test and Bonferroni-Hochberg’s *p-*value correction for multiple comparisons.

In the end, the efficacy of characterization of FNH in multi-phase CT and Gd-BOPTA-enhanced MRI was compared with the implementation of lesions’ localization and readers’ confidence level by means of AFROC analysis. The area under the curve (AUC) for CT and MRI was compared with F-statistics. Statistical analyses were computed with Statistica 12 software (StatSoft Inc., Tulsa, OK, USA) and JAFROC 4.2.1 [12, 14]. A *p* value less than 0.05 was considered significant.

The research was approved by The Independent Bioethics Committee for Scientific Research. All patients gave their informed written consent to participate.

## Results

157 patients who underwent both CT and MRI were included in the analysis.

In 89 patients final diagnosis was based on the histopathological examination: 21 patients were diagnosed with FNH, 35 – HCC, 4 – HCA and 22 with metastases. In the remaining 68 patients, the final diagnosis was based upon clinical and imaging follow-up revealing HH in 36 patients, FNH in 18 patients and liver metastases in 21 patients. In 15 patients diagnosed with FNH, the central scar was seen in histopathological examination. In the rest of FNH specimens areas of congestion were observed.

Table 1 lists all clinical symptoms for non-FNH and FNH group that lead to initial ultrasound examination and subsequently examined in CT and MRI.

**Table 1:** The indication for performing ultrasonography in patients examined by CT and MRI (*n* = 157)

	non-FNH	FNH
digestive tract carcinoma (colorectal/pancreatic/gastric carcinoma)	30/7/1	0/2/0
renal carcinoma	6	1
melanoma malignum	1	0
other neoplasm	6	1*
hepatic cirrhosis	38	0
abdominal pain	13	14
no symptoms	16	21

*breast carcinoma

In 38 patients liver cirrhosis was recognized on the basis of core needle biopsy, 35 of those patients were diagnosed with HCC, one with renal cancer metastasis and two with colorectal cancer metastases.

In FNH-group, slight elevation of serum GGTP level was seen in 10 patients, in all remaining cases biochemical examinations showed no significant changes, none of the patients was diagnosed with cirrhosis. In 6 patients with oncologic history, FLLs were detected at the follow-up abdominal ultrasonography. In 21 cases FNHs were detected incidentally (Table 1).

Patients characteristics in FNH group and non-FNH group are listed in Table 2. The first group included 118 patients with 335 non-FNH foci (Table 3). The second group included 39 subjects with 45 FNH foci.

**Table 2:** Characteristics of the non-FNH and FNH group

	non-FNH	FNH
number of patients	118	39
number of foci	335	45
average age	57	36
male/female ratio	54 / 64	8/31

**Table 3:** Final diagnosis in the non-FNH group

histopathological diagnosis	number of patients	number of foci
HCC	35	95
HH	36	98
metastases	43	138
HCA	4	4

*HCA* Hepatocellular adenoma, *HCC* Hepatocellular carcinoma, *HH* Hepatic hemangioma

Women were significantly more frequent in FNH- group (31/39) than in non-FNH group (64/118), χ^2^ = 7.82, *p* = 0.0052. Statistically significant difference was also seen in patients’ age and lesion diameter: in FNH-group the mean age was 36 years (18÷56) vs 56 years (21÷79) in non-FNH group (Z = 6.97, *p* < 0.0001), in FNH-group the mean diameter of a focus was 37 mm (10÷85 mm) vs 29 mm (5÷80 mm) in non-FNH group (Z = −4.03, *p* < 0.0001).

Interobserver reproducibility and agreement for all parameters were high with Kappa values of 0.85÷1.0.

In analyzed patients, CT revealed 335 lesions and MRI – 380. MRI discovered 21 more HH, 14 more HCC and 10 more metastases. Both in CT and MRI the number of diagnosed FNH foci was equal. 6 patients had bifocal FNH.

**Fig. 2:**
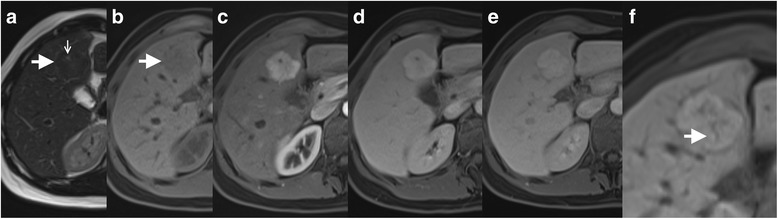
MRI examination of a 33-year-old female; all the images are in the axial plane. Fig. a and b present slightly hypointense FNH in segment IV of the liver (arrow) on T2- and T1-weighted images. Central scar (small arrow) is hyperintense on T2-weighted images (Fig a). In hepatic arterial phase, the lobulated lesion presents strong enhancement (Fig. c). In portal venous phase (Fig. d) and equilibrium phase (Fig. e) the lesion stays hyperintense, however, the enhancement is weaker than in hepatic arterial phase. In hepatobiliary phase (Fig. f) the lesion is hyperintense and enhancement of the scar is visible (arrow)

Radiological signs (Fig. 2 and Additional file 2, Additional file 3 and Additional file 4) appearing statistically more often in FNH than in other FLLs in CT and/or MRI included:

1. homogeneous enhancement in HAP in both CT and MRI (38/40 FNH foci and 67/84 other lesions in CT and MRI relatively),
2. presence of central scar (24/33 FNH foci and 3/5 HH in CT and MRI accordingly),
3. enhancement similar to that of the liver in PVP (36/37 FNH foci and 59/72 other lesions in CT and MRI relatively),
4. enhancement similar to that of the liver in EP (45/45 FNH foci and 164/204 other lesions in CT and MRI relatively),
5. density similar to that of the liver in non-enhanced CT (40 FNH foci and 111 other lesions),
6. isointense or hyperintense signal of the focus in comparison to the adjacent liver parenchyma in HBP (all FNH foci and 21 other lesions),
7. intensity similar to that of the liver on T1- and T2-weighted images in MRI (20 FNH foci and 53 other lesions on T1-weighted images and 38 FNH foci and 49 other lesions on T2-weighted images).

Diagnostic efficacy of above mentioned radiological signs and other analyzed features is presented in Table 4.

**Table 4:** Diagnostic efficiency of radiological findings in CT and MRI and their logical sum

		sensitivity	specificity	PPV	NPV	accuracy
CT	isodense focus in NECT	0.93	0.74	0.36	0.99	0.76
	homogeneous enhancement in HAP	0.84	0.77	0.36	0.97	0.78
	isodense to liver enhancement in PVP	0.80	0.80	0.38	0.96	0.80
	isodense to liver enhancement in EP	1.00	0.43	0.22	1.00	0.51
	central scar	0.53	0.99	0.89	0.93	0.93
	homogeneous enhancement in HAP and central scar	0.47	0.99	0.88	0.93	0.93
	homogeneous enhancement in HAP and isodense to liver enhancement in PVP	0.71	0.95	0.67	0.96	0.92
	homogeneous enhancement in HAP and isodense to liver enhancement in PVP no cirrhosis	0.71	0.97	0.76	0.96	0.94
	homogeneous enhancement in HAP and isodense to liver enhancement in PVP no neoplastic disease in anamnesis	0.55	0.83	0.29	0.94	0.80
MRI	isointense focus in T1 W	0.44	0.84	0.27	0.92	0.79
	isointense focus in T2 W	0.84	0.85	0.44	0.98	0.85
	central scar	0.73	0.99	0.87	0.96	0.96
	homogeneous enhancement in HAP	0.89	0.75	0.32	0.98	0.77
	isointense to liver enhancement in PVP	0.82	0.79	0.34	0.97	0.79
	non-hypointense focus in HBP	1.00	0.94	0.68	1.00	0.94
	homogeneous enhancement in HAP non-hypointense focus in HBP	0.89	0.97	0.82	0.98	0.96
	homogeneous enhancement in HAP non-hypointense focus in HBP central scar	0.64	1.00	1.00	0.95	0.96
	non-hypointense focus in HBP no neoplastic disease in anamnesis	0.55	0.93	0.56	0.92	0.87
	*non-hypointense focus in HBP no cirrhosis*	*1.00*	*0.99*	*0.94*	*1.00*	*0.99*
	homogeneous enhancement in HAP no cirrhosis	0.89	0.83	0.39	0.99	0.84
	homogeneous enhancement in HAP non-hypointense focus in HBP no cirrhosis	0.89	1.00	1.00	0.99	0.99

The logic sum of radiological findings with the highest diagnostic efficiency in italic. *HAP* Hepatic arterial phase, *HBP* Hepatobiliary phase, *NPV* Negative predictive value, *NECT* Non-enhanced computed tomography, PVP Portal venous phase, *PPV* Positive predictive value, *T1 W* T1-weighted images, *T2 W* T2-weighted images

Presence of central scar allows differentiating FNH from other FLLs only in 53% of cases in CT and 73% in MRI (accuracy of 93% and 96% relatively). In both CT and MRI foci with visible scar were significantly larger (mean of 27 mm, SD = 13 mm vs. 45 mm, SD = 12 mm in CT and 20 mm, SD = 9 mm vs. 43 mm, SD = 13 mm in MRI, *p <* 0.001).

Homogeneous enhancement in HAP in both CT and MRI is a feature of a good diagnostic efficacy (78% and 77% accordingly). However, low positive predictive value (PPV) (36% and 32% in CT and MRI accordingly) strongly decreases its clinical usefulness.

Feature characterized by the highest values of sensitivity (100%) and specificity (94%) in the diagnosis of FNH in MRI was the enhancement of the lesion in HBP. In all cases of FNH, the focus was isointense (42 foci) or hyperintense (3 foci) in comparison to the adjacent liver parenchyma in HBP. The same enhancement pattern in HBP as in FNH observed in case of 21 HCC foci (isointense foci) resulted in PPV of 68% for this feature. Moreover, 4 of the enhancing in HBP HCC foci presented with homogeneous enhancement in HAP and were isointense in PVP and EP. However, in all those cases patients suffered from cirrhosis.

To increase the efficacy parameter further analysis was conducted on logic sums of different radiological signs and clinical data. This analysis was performed separately for CT and MRI (Table 4).

Based on MRI study we stated that simultaneous occurrence of two radiological signs (non-hypointense enhancement in HBP and homogeneous enhancement in HAP) was a criterion enabling the diagnosis of FNH with PPV of 82%. Furthermore, after exclusion of cirrhotic patients, the efficacy of HBP reached very high values: sensitivity of 100%, specificity of 99%, PPV of 94%, the negative prognostic value of 100% and accuracy of 100% (Table 4).

By means of Cochran’s Q test, the most efficient radiological signs were compared and significant differences were stated both in CT and in MRI (*p* < 0.001).

In *post hoc* analysis the McNemar’s test with Bonferroni-Hochberg’s correction was performed to compare individual radiological signs and logic sums. Presence of central scar and the logic sum of homogeneous enhancement in HAP and presence of central scar proved to diagnose FNH better than the logic sum of enhancement pattern in HAP and PVP (*p* = 0.0072 and *p* = 0.0006 accordingly). Logic sum of enhancement pattern of HAP and PVP and exclusion of cirrhosis recognized FNH better than the logic sum of homogeneous enhancement in HAP and presence of central scar (*p* = 0.006). However, the logic sum of enhancement pattern of HAP and PVP and exclusion of cirrhotic patients did not perform significantly better than the presence of central scar alone (*p =* 0.054) or the logic sum of enhancement pattern in HAP and PVP (*p =* 0.0824). All the results are presented in Additional file 5. In MRI the logic sum of enhancement pattern in HAP and HBP after exclusion of cirrhotic patients determined FNH better than enhancement pattern in HBP alone (*p* < 0.0001), the logic sum of enhancement pattern in HAP and HBP (*p* < 0.0001) and the logic sum of enhancement pattern in HAP and HBP and presence of central scar (*p* = 0.0037). Though, the logic sum of enhancement pattern in HAP and HBP after exclusion of cirrhotic patients qualified FNH worse than enhancement pattern in HBP after exclusion of cirrhotic patients (*p =* 0.0148). All the results are presented in Additional file 6.

**Fig. 3:**
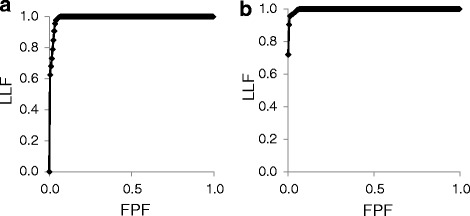
Mean AFROC plot for multi-phase multi-detector CT (**a**) and Gd-BOPTA-enhanced MRI (**b**) in the diagnosis of FNH. LLF – lesion location fraction, FPF – false positive fraction

In AFROC analysis AUC for CT was significantly smaller than for MRI for each reader (Table 5). The mean AUC for CT examination was 0.934 (standard error 0.009, 95% confidence interval 0.9164÷0.951) and for MRI examination 0.941 (SE 0.008, 95% CI 0.924÷0.957), (Fig. 3). The difference between AUCs for CT and MRI reached −0.007 (SE 0.003, 95%CI -0.012÷ −0.001), was statistically significant (*p* = 0.0145) and in favour of MRI examination.

**Table 5:** The area under the curve in AFROC analysis for each reader in CT and MRI

	CT	MRI
1st reader	0.936	0.942
2nd reader	0.933	0.941
3rd reader	0.933	0.939
mean AUC	0.934	0.941

*AUC* Area under the curve

The confidence level of 5 and 4 (very likely and likely) was considered as a positive diagnosis of FNH. In CT the 1st reader rated 33 lesions as FNH (73%, 32 lesions ranged as very likely and single lesion as likely to be FNH), the 2nd reader – 32 lesions (71%, 30 lesions – very likely and 2 lesions – likely) and the 3rd reader – 32 lesions (71%, 31 lesions – very likely and single lesion – likely). In MRI the 1st reader rated 44 lesions as FNH (98%, 37 lesions – very likely and 7 lesions – likely), the 2nd reader – 44 lesions (98%, 32 lesions – very likely and 12 lesions – likely) and the 3rd reader – 41 lesions (91%, 34 lesions – very likely and 7 lesions – likely).

In CT there were six false-negative lesions for the 1st reader (13%), one for the 2nd reader (2%) and two for the 3rd reader (4%, mean of 6.7%). In MRI there were only two false-negative lesions for the 1st reader (4%). The 2nd and 3rd reader diagnosed all FNH lesions correctly (mean 1.5%). All the false-negative lesions were misdiagnosed as HCC.In CT there were four HCC lesions and one metastasis misinterpreted as FNH by the 2nd reader. The 3rd reader also misinterpreted four HCC lesions as FNH. There were no false-positive lesions for the 1st reader. However, only the 1st reader misdiagnosed two HCC lesions and one HCA lesion as FNH in MRI. The misdiagnosed HCA had lobulated shape and showed enhancement in HAP and HBP, therefore mimicking FNH.

In the retrospective analysis, the readers discovered 31 of 41 missed lesions (69%) in CT examinations. The missed lesions were HH (12 lesions), HCC (11) and metastases (8). The decision was based on a consensus of the three readers.

## Discussion

In our study, as well as in available literature, FNH occurs most often in women in the 3rd-5th decades of life [1, 3]. The majority of our cases (70%) were females and mean age was 36 years. In 13% of the patients, FNH was bifocal, and this frequency is similar to the other studies where multi-focal lesions were reported in up to 15% to 20% [3, 15, 16]. FNH is also 10 times more common than HCA [17] and in our study, we diagnosed 4 HCA and 45 FNH. In the group of patients with FNH, over half of the patients were asymptomatic, one-third of the patients complained of abdominal pain, 10% had a neoplastic disease with a primary focus localized outside the liver, none of the patients suffered from liver cirrhosis (Table 1). Biochemical parameters, except for a slight elevation of GGTP activity in 10 patients, did not show any changes. Similar serum GGTP level elevation was also observed by Cherqui et al. [4]. To summarize, the clinical picture of our study group did not differ from the literature data.

In our material, only 53% of lesions showed a central scar in CT. Some authors emphasize that presence of a central scar is typical for FNH foci larger than 3 cm [16, 18]. The relation between the diameter of the lesion and presence of a central scar is confirmed by correlating radiological and microscopic images [19, 20]. Comparable to our study, Brancatelli et al. [16] observed a central scar in CT in half of the subjects in a group of 124 FNH (mean diameter of 41 mm), less often in lesions under 40 mm. In our study mean diameter of FNH with the scar was significantly larger than of foci without visible scar both in CT and MRI. Other authors reported the presence of a central scar in 75% of the cases in HAP in MRI, similar to our study (73%). On the other hand, lack of central scar is an atypical radiological manifestation of FNH, complicating the diagnosis [19, 20]. Our results show that presence of a central scar has high diagnostic specificity, however, the low sensitivity limits its usefulness considerably.

The use of contrast agents allows showing enhancement differences between focal lesions and normal liver parenchyma [5, 6, 8, 9]. Focal lesions such as FNH, hepatocellular carcinoma (HCC) and metastases from kidney or endocrine tumours present abundant vascularization. Most metastases, mainly from the gastrointestinal tract, are typically hypovascular. Generally, evaluation of vascularization of lesions in contrast-enhanced CT and MRI is comparable. In our material, agreement rate of enhancement pattern assessment of particular foci in CT and MRI was 94%. Homogeneous enhancement in HAP was visible in 84% of FHN lesions in CT and 89% in MRI. Those findings concord with results of other authors [21, 22]. Comparable percent of FNH lesions showed enhancement similar to the adjacent liver parenchyma in PVP. However, enhancement pattern in HAP and PVP have very low PPV (32–38%) and the logic sum of the enhancement pattern in HAP and PVP does not improve PPV sufficiently (67%). Moreover, the presence of central scar proved to be a better diagnostic feature than the enhancement pattern in both HAP and PVP. Only after exclusion of cirrhotic patients, diagnostic efficacy of the enhancement pattern in HAP and PVP proved to be comparable with that of a presence of central scar. This data demonstrate a need for more effective diagnostic methods in the differentiation of FNH.

Gd-BOPTA enables detection of lesions containing active hepatocytes. Usefulness of hepato-specific contrast-enhanced MRI in recognition of liver tumours has been well described [6–8].

According to Kim et al., FNH is typically a focus presenting slight enhancement in HBP with a hypointense central scar [21]. In our material, the majority of FNH foci (93%) was isointense with the surrounding liver parenchyma in HBP and only 3 large lesions (≥35 mm) showed a discrete increase of the signal. All FNH foci presented with the enhancement pattern typical for the unchanged hepatocyte and were never hypointense.

In comparison, 4 adenomas, often requiring differentiation with FNH, showed weaker enhancement than the liver parenchyma in HBP what made them unequivocally excluded from further differential diagnosis. A similar observation was made by Grazioli et al. [8] and this is most probably due to the presence of regressive changes in adenomas and lack of bile ducts. However, Roux et al. in the analysis of 27 HCA lesions found 44% of them enhancing in HBP [23]. Our material is too small to compare with that collected by Roux et al.

On the other hand, both in the study of Kim et al. [24] and in ours, malignant lesions (HCC) were found among isointense foci in HBP and constituted 22% of all HCC lesions. Huppertz et al. reported that some highly differentiated HCC can enhance similarly to normal hepatocytes in HBP [25].

The liver-specific phase of MRI in the diagnosis of FNH had satisfactory sensitivity and specificity, only PPV parameter is of 68% (Table 4). However, the sum of two radiological signs: non-hypointense lesion in HBP and homogeneous enhancement in HAP, established as criterion enabling the diagnosis of FNH, was characterized by a higher value of PPV (82%). HCC foci, that show isointensity in HBP and strongly enhance in HAP, are a limitation of Gd-BOPTA-enhanced MRI.

In case of good clinical and radiological cooperation in interdisciplinary teams, improved diagnostics of FLLs is possible with evaluation of additional clinical data (Table 4). Although neoplastic disease in anamnesis is twice more frequent in patients in the non-FNH group, this does not influence differential diagnostics of FNH. However, patients with liver cirrhosis were not suspected of FNH [13]. Finally, after exclusion of cirrhotic patients, presence of non-hypointense (iso- or hyperintense) foci in HBP proved to be the most accurate feature in FNH diagnosis. Parameters of diagnostic efficacy, including PPV, range from 94% to 100% (Table 4). Introduction of these diagnostic parameters gave only three false positive results (non-cirrhotic patient with a small isointense focus in HBP and final diagnosis of HCC). A non-hypointense lesion in HBP after exclusion of cirrhotic patients turned out to diagnose statistically better than other features or their logic sums beside the logic sum of homogeneous enhancement in HAP and a non-hypointense lesion in HBP. According to McNemar’s test, both features diagnosed FNH similarly. However, enhancement pattern in HBP after exclusion of cirrhotic patients showed higher efficacy parameters (Table 4).

In AFROC analysis AUC for MRI was significantly larger than for CT. The authors did not find any other study applying AFROC analysis to compare the efficacy of multi-phase CT and hepatotropic contrast-enhanced MRI in the diagnosis of FNH. Chung et al. compared the efficacy of multi-phase CT and hepatotropic contrast-enhanced MRI in the diagnosis of FFL but assessed only efficacy parameters [26]. Chung et al. concluded that MRI may provide more certain diagnosis, especially in case of FNH. Soussan et al. collected data concerning confidence level of contrast-enhanced ultrasonography in comparison to MRI in the diagnosis of FFLs but did not perform FROC or AFROC analysis [27]. Sofue et al. compared the diagnostic performance of contrast-enhanced CT and a combination of contrast-enhanced CT and hepatotropic contrasted-enhanced MRI with use of AFROC analysis [28] with the conclusion that combination of CT and MRI was more effective than CT alone.

This study has few limitations. Firstly, diffusion-weighted imaging in MRI was not included in the analysis. However, the study was designed to assess the efficacy of morphological and contrast-enhanced examinations only. Secondly, not all lesions were histologically proven. In 43% of patients, the final diagnosis was based upon clinical and radiological follow-up. This group contained all HH cases and almost half of FNH cases, where the biopsy was not possible or necessary. Thirdly, besides HCA the studied group does not include rare entities like fibrolamellar carcinoma, nodular regenerative hyperplasia or cholangiocarcinoma. Although we included incidental findings and the examined group is relatively large, we did not encounter those rare neoplasms. Finally, although we have shown that MRI has higher efficacy in the diagnosis of FNH than CT, one has to take into consideration the cost efficiency of those examinations. This issue goes beyond the scope of this paper.

In the end, it has to be emphasized, that in course of cirrhosis if a new FLL is found, it is unlikely to be FNH. In such a case, even if the lesion presents radiological features of FNH, a liver biopsy should be performed.

## Conclusion

Assessment of dynamic contrast-enhanced MRI with hepatobiliary phase after Gd-BOPTA administration is a useful diagnostic tool. The only limitation of this method is small and/or highly differentiated foci of HCC, strongly enhancing after contrast agent administration in HAP and isointense in HBP. It can be avoided thanks to the cooperation of referring physician and radiologist and excluding patients with known liver cirrhosis. MRI examination with the administration of hepatotropic contrast agent is a more effective method in the differential diagnosis of FNH than multi-phase multi-detector CT. Hepatotropic compounds enable assessment of both vascularization of focal changes and hepatocyte function in the course of one examination (extracellular phase and liver-specific study in the one-stop-shop examination).

## Additional files

Additional file 1: The microscopic image of FNH with characteristic central scar (×20). (TIFF 5111 kb)

Additional file 2: CT and MRI images of FNH in segment IVB of the liver. Fig. a. Axial CT image in hepatic arterial phase shows typical intensive homogeneous enhancement of the lesion with characteristic hypodense central scar (arrow). The lesion is isodense to normal liver parenchyma in the non-contrast examination (Fig. b) and in equilibrium phase (fig. c), but slightly hyperdense in portal venous phase (Fig. d). Fig. e. T1-weighted contrasted-enhanced MRI in hepatic arterial phase shows typical enhancement pattern of FNH - intensive homogeneous enhancement and hypointensive central scar (arrow). Fig. f. Hepatobiliary phase confirms the diagnosis of FNH presenting stronger enhancement of FNH than the surrounding liver parenchyma. This lesion is isointense to the liver parenchyma in non-enhanced T1- (Fig. g) and T2-weighted MRI (Fig. h). (TIFF 528 kb)

Additional file 3: MRI of FNH in segment VI of the liver. Fig. a. Axial T1-weighted non-enhanced MRI shows slightly hypointense focal liver lesion with clearly visible hypointense central scar (arrow). Fig. b. Spin echo sequence, T1-weighted non-enhanced sagittal image presents hypointensive lesion with a central scar. Fig. c. The axial T1-weighted contrast-enhanced image in hepatic arterial phase shows typical intensive homogeneous enhancement of the lesion with a characteristic hypointense central scar. Fig. d. Axial T1-weighted contrast-enhanced MR image: lesion is isointense to the normal liver parenchyma in portal venous phase, the hypointensive central scar is visible (arrow). Fig. e,f. Axial and sagittal images in hepatobiliary phase: lesion is isointense to the surrounding liver parenchyma, the central scar is clearly visible. (TIFF 3002 kb)

Additional file 4: CT and MRI images of FNH in segment V of the liver. Fig. a,b. CT image in hepatic arterial phase shows typical intensive homogeneous enhancement of the lesion with discreetly visible central scar (arrow). This lesion is hypointense in the non-enhanced T1-weighted image (fig. c) and isointense in the T2-weighted image (fig. d). Fig. e. Axial T1-weighted contrast-enhanced MRI in hepatic arterial phase presents a homogeneous enhancement of the lesion with subtle central scar (arrow). (TIFF 909 kb)

Additional file 5: Results of multiple comparisons of radiological signs in CT by means of McNemar’s test. *P* values presented after Bonferroni-Hochberg’s correction. (PDF 108 kb)

Additional file 6: Results of multiple comparisons of radiological signs in MRI by means of McNemar’s test. *P* values presented after Bonferroni-Hochberg’s correction. (PDF 108 kb)

## Abbreviations

AFROC: Alternative free response receiver operating characteristic
AUC: Area under curve
CS: Central scar
CT: Computed tomography
EP: Equilibrium phase
FLL: Focal liver lesion
FNH: Focal nodular hyperplasia
FOV: Field of view
FPF: False positive fraction
Gd-BOPTA: Gadobenate dimeglumine
GGTP: Gamma-glutamyltranspeptidase
HAP: Hepatic arterial dominant phase
HBP: Hepatobiliary phase
HCA: Hepatocellular adenoma
HCC: Hepatocellular carcinoma
HH: Hepatic hemangioma
LLF: Lesion location fraction
MDCT: Multidetector computed tomography
MRI: Magnetic resonance imaging
NECT: Non-enhanced computed tomography
NPV: Negative predictive value
PPV: Positive predictive value
Pts: Patients
PVP: Portal venous dominant phase
ROC: Receiver operating characteristic
SD: Standard deviation
T1 W: T1-weighted images
T2 W: T2-weighted images
TE: Echo time
TR: Repetition time
