# Supplementary figures and images for: Efficacy comparison of multi-phase CT and hepatotropic contrast-enhanced MRI in the differential diagnosis of focal nodular hyperplasia: a prospective cohort study
Source: BMC Gastroenterol. 2018 Jan 15;18:10. doi: 10.1186/s12876-017-0719-1 (PMC5769413; doi:10.1186/s12876-017-0719-1)

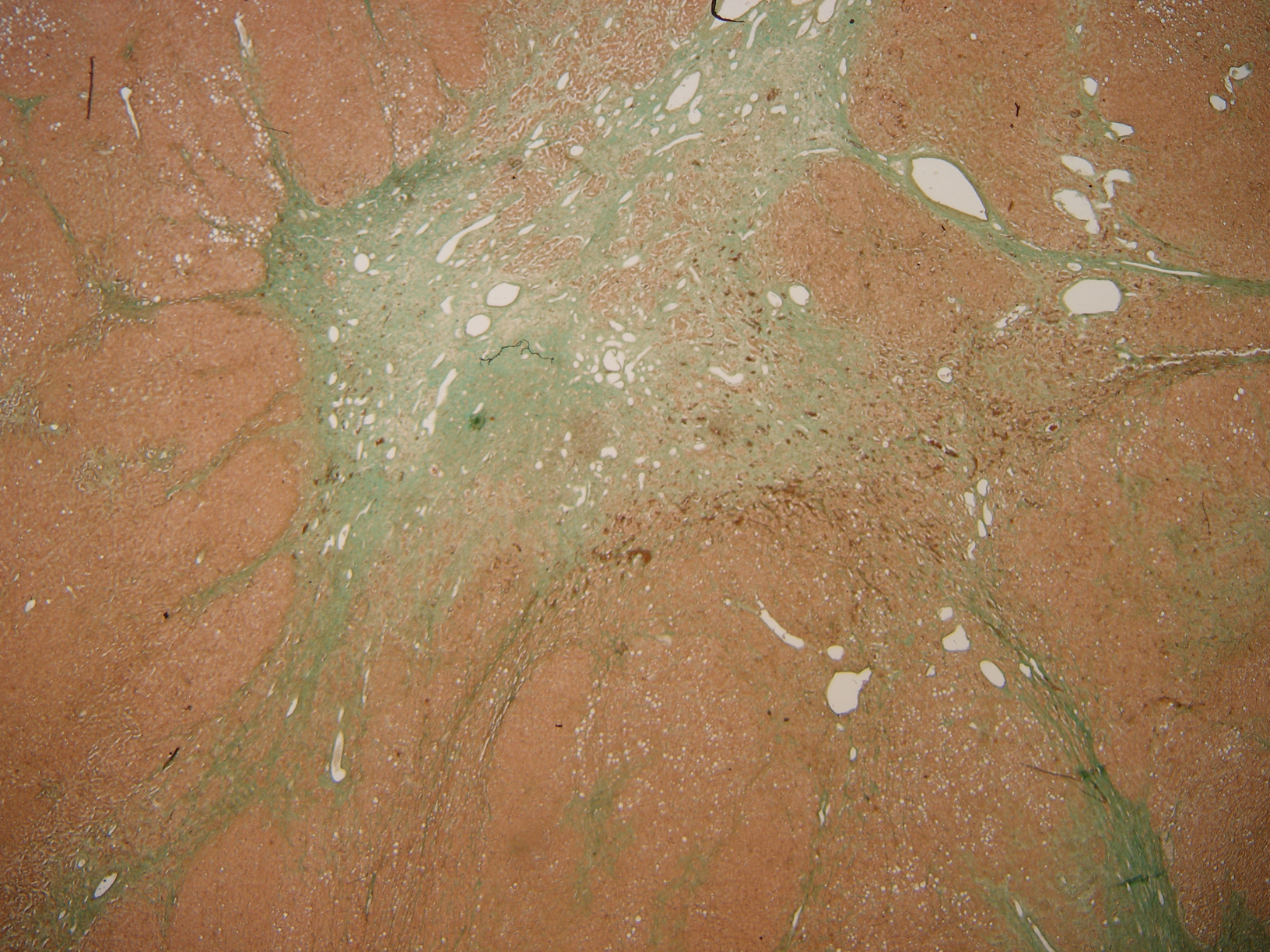

Supplement: Supplementary file 1 — The microscopic image of FNH with characteristic central scar (×20). (TIFF 5111 kb) [file 12876_2017_719_MOESM1_ESM.tif]

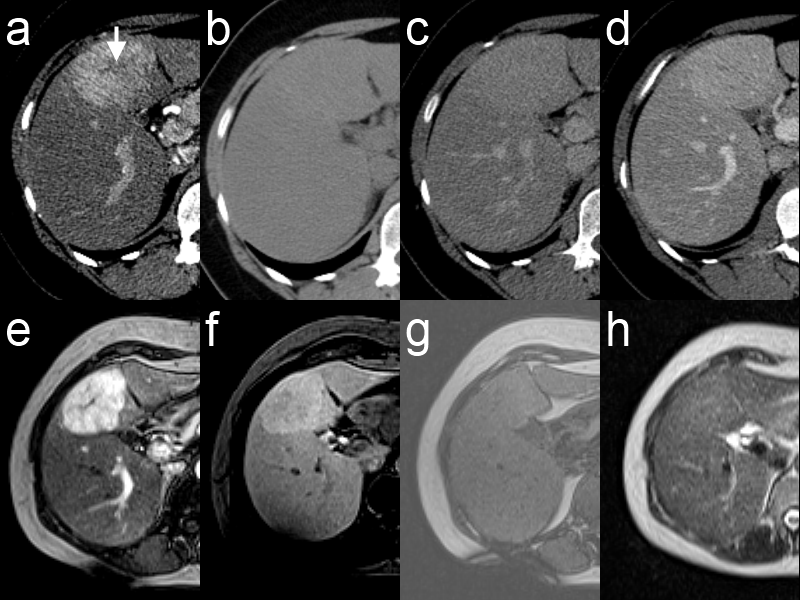

Supplement: Supplementary file 2 — CT and MRI images of FNH in segment IVB of the liver. Fig. a. Axial CT image in hepatic arterial phase shows typical intensive homogeneous enhancement of the lesion with characteristic hypodense central scar (arrow). The lesion is isodense to normal liver parenchyma in the non-contrast examination (Fig. b) and in equilibrium phase (fig. c), but slightly hyperdense in portal venous phase (Fig. d). Fig. e. T1-weighted contrasted-enhanced MRI in hepatic arterial phase shows typical enhancement pattern of FNH - intensive homogeneous enhancement and hypointensive central scar (arrow). Fig. f. Hepatobiliary phase confirms the diagnosis of FNH presenting stronger enhancement of FNH than the surrounding liver parenchyma. This lesion is isointense to the liver parenchyma in non-enhanced T1- (Fig. g) and T2-weighted MRI (Fig. h). (TIFF 528 kb) [file 12876_2017_719_MOESM2_ESM.tif]

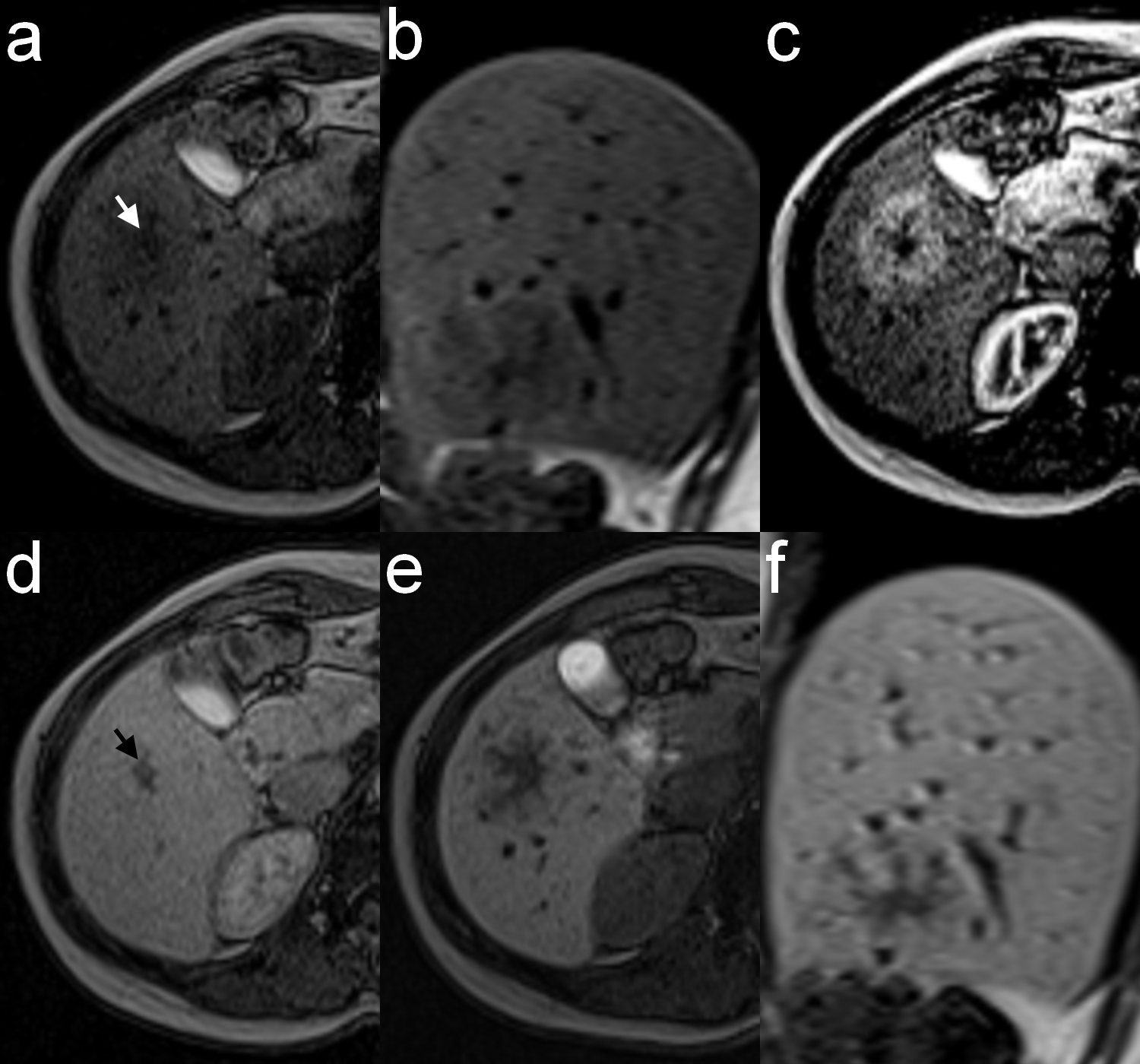

Supplement: Supplementary file 3 — MRI of FNH in segment VI of the liver. Fig. a. Axial T1-weighted non-enhanced MRI shows slightly hypointense focal liver lesion with clearly visible hypointense central scar (arrow). Fig. b. Spin echo sequence, T1-weighted non-enhanced sagittal image presents hypointensive lesion with a central scar. Fig. c. The axial T1-weighted contrast-enhanced image in hepatic arterial phase shows typical intensive homogeneous enhancement of the lesion with a characteristic hypointense central scar. Fig. d. Axial T1-weighted contrast-enhanced MR image: lesion is isointense to the normal liver parenchyma in portal venous phase, the hypointensive central scar is visible (arrow). Fig. e,f. Axial and sagittal images in hepatobiliary phase: lesion is isointense to the surrounding liver parenchyma, the central scar is clearly visible. (TIFF 3002 kb) [file 12876_2017_719_MOESM3_ESM.tif]

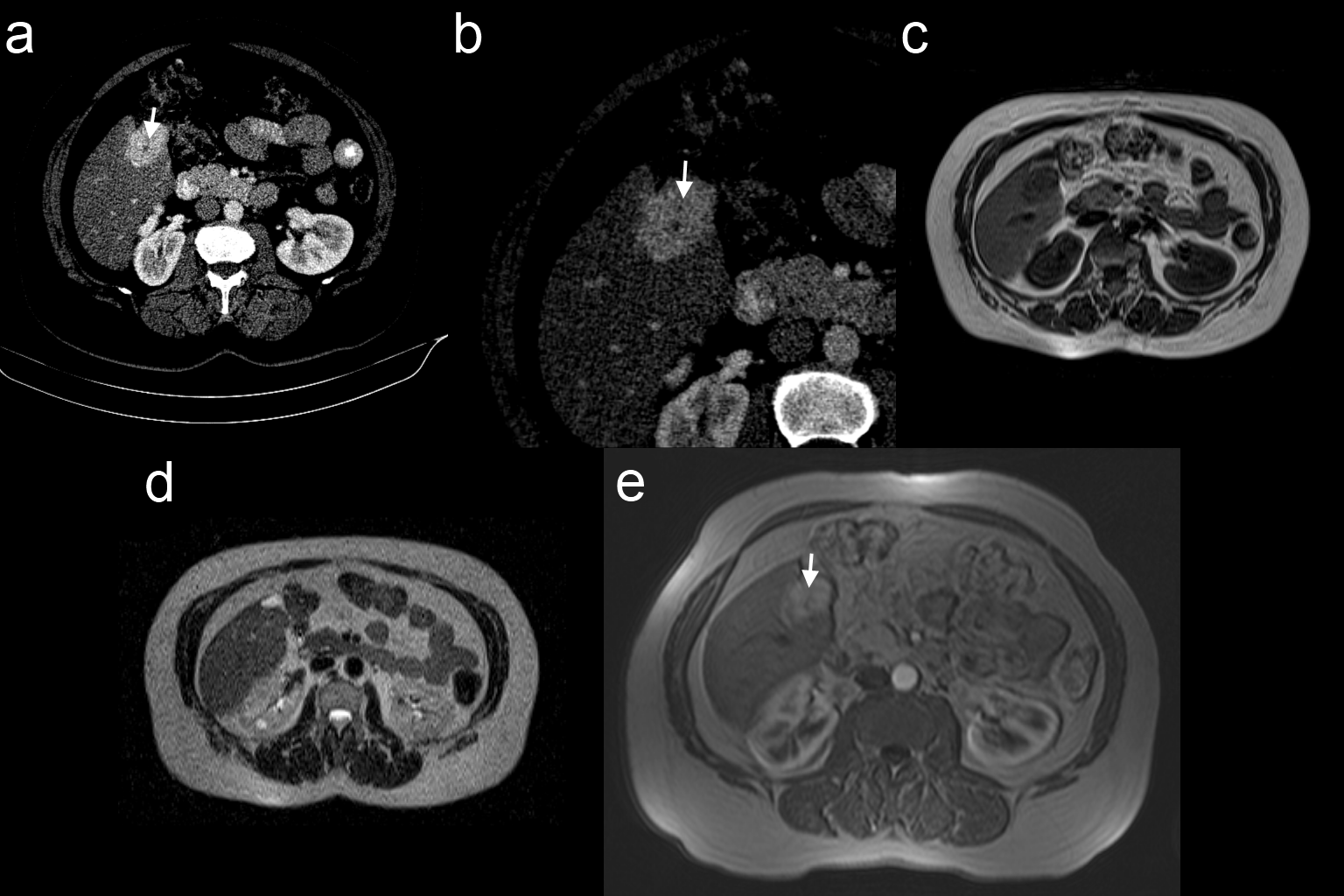

Supplement: Supplementary file 4 — CT and MRI images of FNH in segment V of the liver. Fig. a,b. CT image in hepatic arterial phase shows typical intensive homogeneous enhancement of the lesion with discreetly visible central scar (arrow). This lesion is hypointense in the non-enhanced T1-weighted image (fig. c) and isointense in the T2-weighted image (fig. d). Fig. e. Axial T1-weighted contrast-enhanced MRI in hepatic arterial phase presents a homogeneous enhancement of the lesion with subtle central scar (arrow). (TIFF 909 kb) [file 12876_2017_719_MOESM4_ESM.tif]
