# Supplementary material for: Efficacy comparison of multi-phase CT and hepatotropic contrast-enhanced MRI in the differential diagnosis of focal nodular hyperplasia: a prospective cohort study
Source: BMC Gastroenterol. 2018 Jan 15;18:10. doi: 10.1186/s12876-017-0719-1 (PMC5769413; doi:10.1186/s12876-017-0719-1)
Supplement: Supplementary file 5 — Results of multiple comparisons of radiological signs in CT by means of McNemar’s test. P values presented after Bonferroni-Hochberg’s correction. (PDF 108 kb) [file 12876_2017_719_MOESM5_ESM.pdf]

Additional file 5. Results of multiple comparisons of radiological signs in CT by means of McNemar's test. *P* values presented after Bonferroni-Hochberg's correction.

| | enhancement in HAP and presence of CS | enhancement in HAP and PVP | enhancement in HAP and PVP after exclusion of cirrhotic pts. |
|---|---|---|---|
| presence of CS | 0.134 | **0.0072** | 0.054 |
| enhancement in HAP and presence of CS | | **0.0006** | **0.006** |
| enhancement in HAP and PVP | | | 0.0824 |

CS – central scar, HAP – hepatic arterial phase, pts – patients, PVP – portal venous phase.
