# Supplementary material for: Efficacy comparison of multi-phase CT and hepatotropic contrast-enhanced MRI in the differential diagnosis of focal nodular hyperplasia: a prospective cohort study
Source: BMC Gastroenterol. 2018 Jan 15;18:10. doi: 10.1186/s12876-017-0719-1 (PMC5769413; doi:10.1186/s12876-017-0719-1)
Supplement: Supplementary file 6 — Results of multiple comparisons of radiological signs in MRI by means of McNemar’s test. P values presented after Bonferroni-Hochberg’s correction. (PDF 108 kb) [file 12876_2017_719_MOESM6_ESM.pdf]

Additional file 6. Results of multiple comparisons of radiological signs in MRI by means of McNemar’s test. *P* values presented after Bonferroni-Hochberg’s correction.

	enhancement in HAP and HBP	enhancement in HAP and HBP and presence of CS	enhancement in HBP after exclusion of cirrhotic pts.	enhancement in HAP and HBP after exclusion of cirrhotic pts
enhancement in HBP	**0.0002**	**<0.0001**	**0.0002**	**<0.0001**
enhancement in HAP and HBP		**<0.0001**	0.8137	**0.0096**
enhancement in HAP and HBP and presence of CS			**<0.0001**	**0.0037**
enhancement in HBP after exclusion of cirrhotic pts.				**0.0148**

CS – central scar, HAP – hepatic arterial phase, HBP – hepatobiliary phase, pts – patients.
